# Two new species of *Trichomerium* (Trichomeriaceae, Chaetothyriales) from Guizhou Province, China

**DOI:** 10.3897/mycokeys.137.177643

**Published:** 2026-07-22

**Authors:** Xiaolu Deng, Yuwei Liu, Sinang Hongsanan, Tian Yang, Yuzhe Feng, Jipeng Sun, Xiang-Yu Zeng

**Affiliations:** 1 Department of Plant Pathology, College of Agriculture, Guizhou University, Guiyang, 550025 China College of Life Science and Oceanography, Shenzhen University Shenzhen China; 2 Guizhou Key Laboratory of Agricultural Microbiology, Guiyang, 550025 China College of Agriculture, Guizhou University Guiyang China; 3 Institute of Edible Mushrooms, Guizhou University, Guiyang, 550025 China Institute of Edible Mushrooms, Guizhou University Guiyang China; 4 Shenzhen Key Laboratory of Microbial Genetic Engineering, College of Life Science and Oceanography, Shenzhen University, Shenzhen 518060, China Guizhou Key Laboratory of Agricultural Microbiology Guiyang China

**Keywords:** *
Eurotiomycetes
*, morphology, phylogeny, sooty mold, taxonomy

## Abstract

*Trichomerium* is a genus of foliar epiphytic fungi that resemble sooty molds and are commonly found on the surfaces of living leaves. Species within this genus are characterized by dark, intricately interwoven hyphae and ascomata bearing setae. Two novel species forming sooty coating colonies, namely *Trichomerium guiyangense* and *T. xishuiense*, were discovered in Guizhou Province, China. These taxa are morphologically distinct and were formally identified through comprehensive morphological characterization and multi-gene phylogenetic analyses based on two genetic loci (LSU and ITS rDNA). The taxonomic descriptions are supported by detailed illustrations and a phylogenetic tree elucidating the placement of the newly described species. This study enhances the understanding of the taxonomic diversity within *Trichomerium* and refines its species delimitation and systematics by contributing significant morphological and molecular evidence, expanding the known diversity of this genus in China, and providing new insights into its phylogeny and taxonomy.

## Introduction

Trichomeriaceae is a recently established lineage within the order Chaetothyriales (Chomnunti et al. 2012a). Trichomeriaceae has been reported to cause plant diseases, and some species are recorded as opportunistic pathogens of humans and animals. Subsequent taxa of Trichomeriaceae were also isolated from rock surfaces and architectural relics and were summarized as rock-inhabiting fungi (Nascimento et al. 2016; Sun et al. 2020). Members of Trichomeriaceae display a diverse range of ecological adaptations, with species inhabiting various environments but particularly showing high abundance in tropical rainforests (Hyde et al. 2020). The family currently accommodates multiple genera, including *Anthracina*, *Arthrocladium*, *Bradymyces*, *Formicomyces*, *Incumbomyces*, *Knufia*, *Lithohypha*, *Neostrelitziana*, *Pararthrophiala*, *Pasadenomyces*, *Strelitziana*, and *Trichomerium* (Sun et al. 2020; Hyde et al. 2024).

*Trichomerium*, the type genus of Trichomeriaceae, is commonly found as foliar epiphytic sooty moulds on living leaves (Hongsanan et al. 2016; Rana et al. 2019). The genus was introduced by Spegazzini (1918) with *T. coffeicola* as the type species. Its taxonomic placement has undergone significant revisions: it was initially classified in Capnodiaceae (Batista and Ciferri 1963), later transferred to Triposporiopsidaceae (Hughes 1976), and then to Chaetothyriaceae based on morphological data (Chomnunti et al. 2012b). Morphologically, *Trichomerium* differs from species in Capnodiaceae and Chaetothyriaceae by having an apical ascal ring, ascospores with or without sheaths, and unique septation patterns. Besides, *Trichomerium* exhibits both sexual and asexual morphs. The sexual morph is characterized by superficial, setiferous, uniloculate ascomata that are surrounded by loosely interwoven mycelium and which produce bitunicate asci and hyaline, septate ascospores (Chomnunti et al. 2012a; Hongsanan et al. 2016). The asexual morph of *Trichomerium* is notably characterized by its conidia, which are typically dark, multi-segmented, and exhibit a distinctive star-like structure with multiple radiating arms (Crous et al. 2014; Hongsanan et al. 2016; Sun et al. 2020). Regarding molecular characteristics, phylogenetic analyses indicate that *Trichomerium* species form a distinct cluster separate from both Capnodiaceae and Chaetothyriaceae (Chomnunti et al. 2012a, 2012b; 2014; Hongsanan et al. 2016). Consequently, *Trichomerium* is currently accepted in the family Trichomeriaceae within the order Chaetothyriales.

Index Fungorum lists 34 records of *Trichomerium*, with sequence data available on NCBI for 21 species, with *Pasadenomyces* represented by a whole genome sequence (accessed 25 June 2025). The database contains a large number of names, but the actual number of valid species is far fewer than the number of names (Yang et al. 2021). Moreover, even fewer species have molecular data, indicating that taxonomic studies of this genus still require further refinement. This highlights the urgent need for further taxonomic and ecological research to fill these gaps in our knowledge.

Several species of *Trichomerium* have been reported from China, demonstrating its existing diversity in the country. For example, *T. cicatricatum*, *T. flexuosum*, and *T. lapideum* were described from rock substrates in northern China (Sun et al. 2020), and *T. leigongense* represents a rock-inhabiting lineage recorded from Guizhou Province (Sun et al. 2020). In southwestern China, *T. multisetosum*, *T. xishuangbannaense*, and *T. yunnanense* were identified as sooty moulds in Yunnan Province (Yang et al. 2021). Despite these records, the diversity of foliar epiphytic *Trichomerium* (sooty moulds) in Guizhou Province remains poorly investigated. This study introduces two new species of *Trichomerium* collected from Guizhou Province, China. The discovery of these species enhances our understanding of the diversity and distribution of *Trichomerium* in China and provides valuable taxonomic and ecological insights for the genus. These new species are described based on detailed morphological characterization and supported by phylogenetic analyses of LSU and ITS sequence data.

## Materials and methods

### Fungal sampling, isolating, and morphology

Leaves exhibiting symptoms of black mildew infection were collected from Aha Lake Wetland Park, Guiyang City, and Xishui, Guizhou Province. These samples were carefully transported to the laboratory in paper envelopes and maintained in a dry setting to preserve their integrity. Pure cultures were obtained using a single-spore isolation method as described in Chomnunti et al. (2014). A single ascoma was submerged in 300 µL of sterile distilled water on a microscope slide and allowed to sit for several minutes to facilitate the release of ascospores. Subsequently, an ascospore suspension was prepared, and small droplets were placed onto water agar (WA) plates. The plates were then incubated at ambient temperature for 8–12 hours to allow ascospore germination. Once germinated, individual ascospores were transferred to potato dextrose agar (PDA) plates, which were subsequently incubated at 25 °C for 15–20 days. After approximately one month, when the colonies nearly covered the entire plate, the resultant cultures were used for molecular analyses and preserved in 5% glycerol.

Macroscopic features were observed and photographed using a Keyence VHX–7000 digital stereomicroscope. Diagnostic morphological features were recorded in photomicrographs, and measurements were taken. The setae and conidiomata were immersed in water and observed under a Zeiss Axioscope 5 compound microscope (Jena, Germany). They were photographed using an AxioCam 208 color camera (Jena, Germany) and saved as JPG files. Multiple measurements of each feature were made using ZEN 2.0 (blue edition) software. Images used for figures were processed with Adobe Photoshop 2022 software. Type specimens were deposited in the Herbarium of the Department of Plant Pathology, Agricultural College, Guizhou University (**HGUP**). Ex-type cultures were deposited in the Culture Collection at the Department of Plant Pathology, Agricultural College, Guizhou University, P.R. China (**GUCC**).

### Registration of names

New species were registered at Index Fungorum (https://www.indexfungorum.org) and obtained identifiers.

### DNA extraction, PCR amplifications, and sequencing

Total genomic DNA was extracted directly using a DNA extraction kit (Biomiga, San Diego, CA, USA). For nucleotide sequence comparisons, the internal transcribed spacer region (ITS) and a segment of the large subunit rDNA (LSU) were amplified using primer pairs ITS4/ITS5 (White et al. 1990) and LR0R/LR5 (Rehner and Samuels 1994; Vilgalys and Hester 1990). Polymerase chain reactions (PCR) were performed in a 20 µL reaction mixture containing 17 µL of GoldenStar T6 Super PCR Mix (1.1×), 1 µL of DNA template, and 1 μL each of forward and reverse primers (10 μm/µL). The PCR reaction procedures for different primers are shown in Table 1. PCR amplification products were assayed via electrophoresis in 1% (w/v) agarose. The PCR products were sent to Tsingke Biotechnology Co., Ltd., Beijing, China. Newly generated sequences were deposited in GenBank. All taxa used in the phylogenetic analyses are listed in Table 2.

**Table 1.** Molecular markers, PCR primers, and amplification programs used in this study.

**Loci**	**Primers**	**Sequence (5’–3’)**	**PCR Cycles**	**References**
ITS	ITS1	TCCGTAGGTGAACCTGCGG	94 °C: 5 min, (94 °C: 30 s, 50 °C: 1 min, 72 °C: 30 s) × 35 cycles, 72 °C: 10 min	White et al. (1990)
	ITS4	TCCTCCGCTTATTGATATGC		
LSU	LR0R	ACCCGCTGAACTTAAGC	94 °C: 5 min, (94 °C: 30 s, 53 °C: 1 min, 72 °C: 30 s) × 35 cycles, 72 °C: 10 min	Rehner and Samuels (1994); Vilgalys and Hester (1990)
	LR5	TCCTGAGGGAAACTTCG		

**Table 2.** GenBank accession numbers of DNA sequences used in this study.

**Species**	**Strain number**	**GenBank accession number**		**References**
		**ITS**	**LSU**	
*Anthracina ramosa*	CGMCC 3.16372	NR_172152	KP174923	Sun et al. (2020)
*Arthrocladium caudatum*	CBS 457.67	NR_145398	NG_057084	Nascimento et al. (2016)
*Bradymyces alpinus*	CCFEE 5493	NR_132844	NG_058641	Ruibal et al. (2009)
*Formicomyces microglobosus*	WA 0000145466	ON972664	NG_243734	Siedlecki et al. (2023)
*Incumbomyces delicatus*	CBS 128958	NR_176739	–	Quan et al. (2021)
*Knufia victoriae*	CBS 149015	NR_185660	NG_228911	Tsuneda et al. (2011)
*Lithohypha guttulata*	CCFEE 5885	KP791774	–	Crous et al. (2015)
*Neostrelitziana acaciigena*	CBS 139903	NR_137987	NG_058165	Crous et al. (2015)
*Pararthrophiala adonis*	CBS 150825	NR_197927	NG_244050	Crous et al. (2024)
*Phaeosaccardinula ficus*	MFLUCC 10-0009	NR_132850	NG_059455	Yang et al. (2014)
*Phaeosaccardinula multiseptata*	IFRDCC 2639	NR_132894	–	Yang et al. (2014)
*Strelitziana malaysiana*	CBS 139902	KR476731	KR476766	Crous et al. (2015)
*Trichomerium bambusae*	MFLU 16-2286	NR_155921	–	Hyde et al. (2016)
*Trichomerium bhatii*	NFCCI 4305	NR_182452	NG_153865	Rana et al. (2019)
*Trichomerium camporesii*	MFLU 19-2251	NR_169997	MN644511	Hyde et al. (2020)
*Trichomerium chiangmaiensis*	SQUCC 12166	NR_169936	NG_068833	Maharachchikumbura et al. (2018)
*Trichomerium cicatricatum*	CGMCC 3.17307	NR_172232	NG_075200	Sun et al. (2020)
*Trichomerium deniqulatum*	MFLUCC 10-0884	NR_132965	NG_059479	Chomnunti et al. (2011)
*Trichomerium dioscoreae*	CBS 138870	NR_137946	NG_058126	Liu et al. (2014)
*Trichomerium eucalypti*	CBS 143443	NR_156672	NG_058525	Crous et al. (2017)
*Trichomerium flexuosum*	CGMCC 3.17988	NR_172253	NG_075211	Su et al. (2015)
*Trichomerium foliicola*	MFLUCC 10-0078	NR_144963	JX313661	Chomnunti et al. (2012a)
*Trichomerium gleosporum*	MFLUCC:15-0209	KY381954	JX313662	Chomnunti et al. (2012a)
*Trichomerium guiyangense*	GUCC 23-0130	PQ871489	PQ871492	This study
*Trichomerium lapideum*	CGMCC 3.17311	NR_172231	NG_074886	Sun et al. (2020)
*Trichomerium leigongense*	CGMCC 3.17983	NR_172254	KX348471	Sun et al. (2020)
*Trichomerium multisetosum*	IFRDCC 3300	MZ822135	MZ822132	Yang et al. (2021)
*Trichomerium siamensis*	MFLUCC 12-0105	KP744468	–	Ariyawansa et al. (2015)
*Trichomerium koreanum*.	KNUF-23-13A	PP907030	PP907029	Lim et al. (2024)
*Trichomerium syzygii*	CPC 37184	NR_170066	MT223936.1	Crous et al. (2020)
*Trichomerium wuzetian*	BRIP 71373a	NR_189978	–	Crous et al. (2023)
*Trichomerium xishuangbannaense*	IFRDCC 3301	MZ822134	MZ822131	Yang et al. (2021)
*Trichomerium xishuiense*	GUCC 23-0131	PQ871491	PQ871494	This study
*Trichomerium yunnanense*	IFRDCC 3302	MZ822136	MZ822133	Yang et al. (2021)

“–” indicates missing data.

### Phylogenetic analyses

Consensus sequences were assembled from forward and reverse primer reads using BioEdit v. 7.2.5 (Hall 1999). The initial identification of species was conducted by searching against sequences in the NCBI BLASTn search engine (https://blast.ncbi.nlm.nih.gov/Blast.cgi). Phylogenetic analysis was conducted based on datasets including reference DNA sequences and newly generated DNA sequences using OFPT (Zeng et al. 2023) with the following protocol. Initially, sequence downloading: reference sequences were downloaded from the NCBI database. Subsequently, sequence alignment was performed using MAFFT v7 (Kazutaka et al. 2002) with the ‘auto’ strategy, which employs Fast Fourier Transform and iterative refinement for optimal alignment. Poorly aligned regions that could potentially introduce noise into the analysis were then identified and excluded using TrimAl (Capella-Gutierrez et al. 2009) with trimming mode ‘-gappyout’. Model selection was performed using ModelFinder (Kalyaanamoorthy et al. 2017), which evaluated multiple common DNA substitution models with rate heterogeneity. The best-fit model for each gene was determined based on the Bayesian information criterion (BIC). Bayesian inference employed two parallel Markov chain Monte Carlo (MCMC) runs, each with four chains (one cold, three heated). Sampling occurred every 100 generations, with diagnostics every 1,000 generations and checkpoints every 10,000 generations. Analyses were terminated when the split frequency standard deviation fell below 0.01, and consensus trees were generated after a 25% burn-in. Maximum likelihood analyses were conducted in IQ-TREE 2 (Quang et al. 2020) with the best-fit model and 1,000 ultrafast bootstrap replicates (Hoang et al. 2017).

## Results

### Phylogenetic analysis

**Figure 1.**
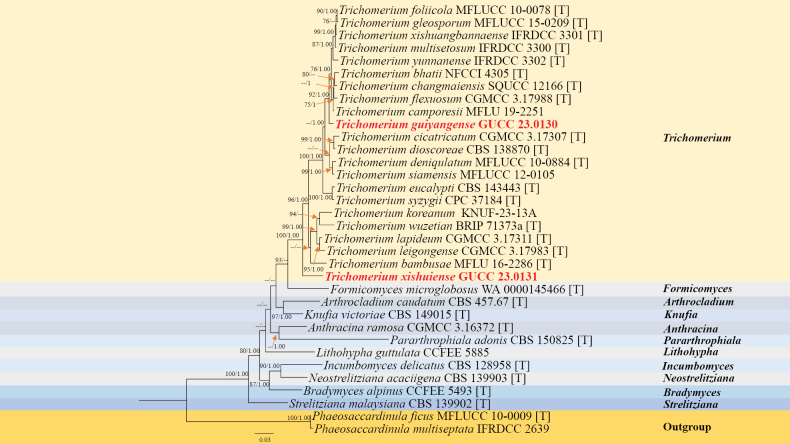
Phylogram of Trichomeriaceae reconstructed from a two-locus dataset (ITS, LSU) using ML and BI algorithms. The Bootstrap support (BS) values greater than 70% and Bayesian posterior probabilities (PP) greater than 0.9 are shown above the branches (ML/PP). *P. ficus* (MFLUCC 10-0009) and *P. multiseptata* (IFRDCC 2639) were selected as outgroups. Sequences obtained from ex-type strains are marked with “T.” New sequences obtained in this study are indicated in bold red.

Phylogenetic trees were constructed using combined LSU and ITS sequences, which comprised 34 species representing the family Trichomeriaceae (Table 2), with *Phaeosaccardinula ficus* (MFLUCC 10-0009) and *P. multiseptata* (IFRDCC 2639) designated as outgroups based on recent taxonomic treatments of Chaetothyriales (Yang et al. 2014). The best nucleic acid substitution models for Bayesian inference of the two gene fragments are as follows: ITS: TNe+I+G4; LSU: TNe+R2. Based on the concatenated ITS–LSU sequence (ITS: 1–449; LSU: 450–1466), the ITS sequences comprised a total of 449 characters, including 233 constant and invariant sites, 169 parsimony-informative sites, and 218 distinct site patterns. The LSU sequences comprised a total of 1,017 characters, including 819 constant sites, 101 parsimony-informative sites, and 237 distinct site patterns. The combined dataset yielded a best-scoring tree with a final ML optimization likelihood value of –7620.213435. The topology of the tree from the Bayesian analysis is similar to the one obtained from the maximum likelihood analysis. Thus, only the ML tree is presented (Fig. 1).

Based on the ML and BI analyses, the genus Trichomeriaceae comprises twelve distinct lineages, which are represented by the genera *Anthracina*, *Arthrocladium*, *Bradymyces*, *Formicomyces*, *Incumbomyces*, *Knufia*, *Lithohypha*, *Neostrelitziana*, *Pararthrophiala*, *Pasadenomyces*, *Strelitziana*, and *Trichomerium*. Notably, *Trichomerium* and *Formicomyces* are sister genera.

### Taxonomy

#### Trichomerium guiyangense

Taxon classificationFungiChaetothyrialesTrichomeriaceae

X.L. Deng & X.Y. Zeng sp. nov.

2E2F053D-ED75-50C5-8D45-B328D6B08B2B

Fig. 2

##### Etymology.

In reference to the location where the specimen was collected

**Figure 2.**
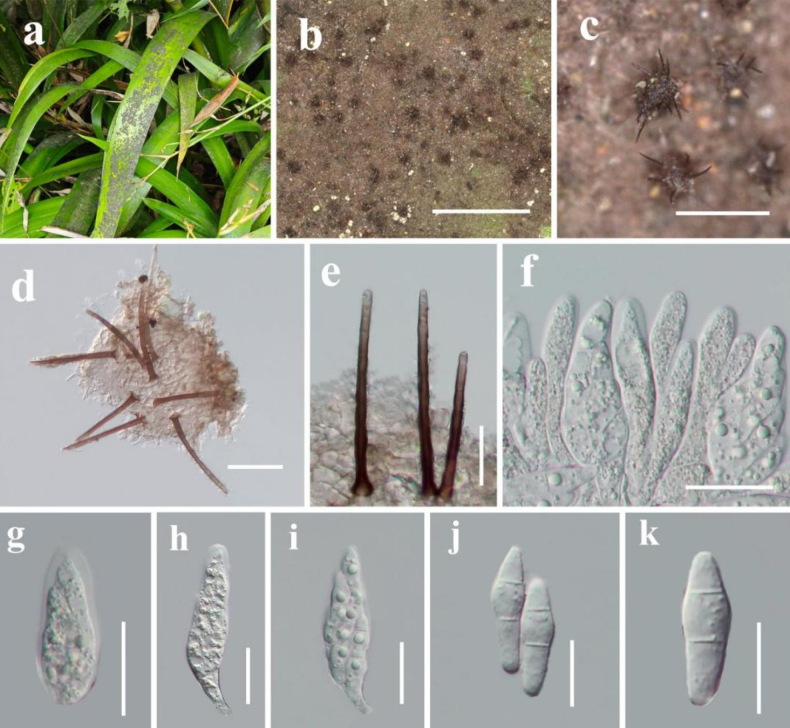
*Trichomerium guiyangense* (GUCC 23-0130). **a, b**. Sooty moulds on leaf surface; **c–e**. Ascomata with setae; **f–i**. Asci; **j, k**. Ascospores. Scale bars: 1000 µm (**b**), 200 µm (**c**), 50 µm (**d**), 20 µm (**e–k**).

##### Description.

Black sheets of superficial mycelia cover the surface of leaves of the host. Hyphae cylindrical, light brown to brown, septate. **Sexual morph**. Ascomata up to 260.4 µm in diameter, subglobose to globose, black, sessile, with an ostiole, with scattered setae on the upper surface. Setae 62.8–86.6 µm long (x̄= 76.5, n = 10), dark brown or olivet, aseptate, straight. Asci 45.5–54.1 × 10.4–18.2 µm (x̄= 49.8 × 14.5 µm, n = 10), bitunicate, 8-spored, cylindrical to clavate. Ascospores 14–18.3 × 4.3–5.6 µm (x̄= 17.2 × 4.8 µm, n = 10), oblong-ellipsoid, slightly narrow and blunt ends, widest at the center, hyaline, overlapping 2–3 seriate, 2-transverse septa, constricted at septa, with sparse guttules, without mucous sheath. **Asexual morph**. Undetermined.

##### Material examined.

China • Guizhou Province, Guiyang City, 26°36'14"N, 106°41'40"E, on living leaves of *Iris* sp., 15 May 2023, Yuwei Liu, QLS42 (HGUP 230079, holotype), ex-type, GUCC 23-0130

##### Notes.

*Trichomerium guiyangense* has morphological characteristics similar to those of other species in the genus *Trichomerium*, such as ascomata with setae and bitunicate asci with hyaline ascospores. In the phylogenetic analysis, the strain GUCC 23-0130 forms a distinct clade with high node support (ML/BI = 92/1), and from the topology, it is phylogenetically distant from neighboring species (Fig. 1). BLAST results revealed nucleotide differences between *T. guiyangense* and its relatives, showing 10% (499/555 nucleotides, 5 gaps) in ITS and 2% (798/811 nucleotides, 0 gaps) in LSU compared to *T. cicatricatum*, and 4% (553/575 nucleotides, 9 gaps) in ITS and 2% (804/818 nucleotides, 0 gaps) in LSU compared to *T. camporesii*. Morphologically, *T. guiyangense* differs from closely related species by its large ascomata (up to 196 µm diameter), and it differs from *T. bhatii* by its 2-seriate (vs. 3-seriate) ascospores (Table 3). Other species different from this species include *T. cicatricatum*, which only exhibits an asexual morph, and *T. guiyangense*, which possesses a sexual morph. Compared to *T. camporesii*, this species has larger asci (45.5–54.1 × 10.4–18.2 µm vs. 55–60 × 20–25 µm) and smaller ascospores (14–18.3 × 4.3–5.6 µm vs. 25–30 × 5–10 µm) (Hyde et al. 2020) (Table 3). Hence, based on both morphology and phylogeny, we identify our specimens as a new species.

**Table 3.** Main sexual morphological characteristics of Trichomerium mentioned in this study.

**Species**	**Ascomata**	**Setae**	**Asci**	**Ascospores**	**References**
*T. abhorrens*	–	95–150 µm long	Elliptical, apedicellate, 50–65 × 22.5–27.5 μm	Fusoid, 3-septate, with mucilaginous sheath, 27.5–35 × 7.5–10 μm	Batista (1951)
*T. bambusae*	140–180 × 105–130 μm	Apical, slightly narrower at the apex, 50–80 × 5–8 μm	Broadly cylindrical or oblong, short-pedicellate, bitunicate, 53–62 × 19–24 μm	Oblong, 3-septate, with mucilaginous sheath, 15–18 × 5–7 μm	Hyde et al. (2016)
*T. camporesii*	–	–	Ellipsoid to clavate, some subglobose, with or without short pedicle, bitunicate, 55–60 × 20–25 μm	Globose-subglobose, brown, 55–65 × 45–55 μm	Hyde et al. (2020)
*T. coffeicola*	70–95 μm in diam.	Simple, continuous, long, and tapered	–	Subfusoid, 2-septate, 3-guttules, 15–18 × 5–6 μm	Batista and Ciferri (1963)
*T. crotonis*	50–65 μm in diam.	–	Sessile, 37.5–50 × 17.5–25 μm	Cylindrico-fusoid, not constricted, 19.8–28 × 6.63–7.5 μm	Batista (1951)
*T. deniqulatum*	154–175 × 163–180 μm	Sparse, indistinct, 32–54 × 3.7–6 μm	Ellipsoid to clavate, some subglobose, bitunicate, 47–60 × 22–31 μm	Fusoid, 3-septate, constricted, 18–25 × 6–8 μm	Chomnunti et al. (2012a)
*T. didymopanacis*	105–150 μm in diam.	Straight or curved, numerous, 50–130 × 5–8 μm	–	1–3 septate, 16–24.5 × 5–7.5 μm	Chomnunti et al. (2012a)
*T. foliicola*	133–179 × 140–181 μm	Abundant, straight, (44–)61–118 × 4–7 μm	Ellipsoid to clavate, some obovoid, bitunicate, (47–)63–70 × (14–)20–26 μm	Fusoid, 2–3 septate, 19–22 × 6–7 μm	Chomnunti et al. (2012a)
*T. gloeosporum*	116–140 × 113–150 μm	Abundant, straight, 71–121 × 4–7 μm	Ellipsoidal to cylindrical, with short pedicel, apparently bitunicate, 62–86 × 18–23 μm	Fusoid, 2–3-septate, with mucilaginous sheath, not constricted, 17–26 × 5–7 μm	Chomnunti et al. (2012a)
*T. grandisporum*	75–102 × 91–160 μm	107–130 × 4–7 μm	–	Fusiform to elliptical, 3-septate, 18.6–24.12 μm	Kwee (1988)
*T. deniqulatum*	154–175 × 163–180 μm	Sparse, indistinct, 32–54 × 3.7–6 μm	Ellipsoid to clavate, some subglobose, bitunicate, 47–60 × 22–31 μm	Fusoid, 3-septate, constricted, 18–25 × 6–8 μm	Chomnunti et al. (2012a)
*T. guiyangense*	196 µm in diam.	Straight, aseptate, constricted 62.8–3.9 × 86.6–7 µm	Cylindrical to clavate, bitunicate, 45.5–54.1 × 10.4–18.2 µm	Fusoid, 2–3-septate, constricted, 14.9–18.3 × 4.3–5.6 μm	This study
*T. hirtellum*	90–150 μm in diam.	Sparse, septate to continuous, black, 56–65 × 5–7.5 μm	Cylindrical or cylindric-clavate, short-pedicellate, 56–78.4 × 8.4–14 μm	Fusoid, 2-septate, 14.5–19.5 × 6–8.5 μm	Batista (1951)
*T. multisetosum*	116–130 × 91–118 μm	Straight, sparse, indistinct, aseptate to septate, (28–)32–54 × 3.7–6 μm	Ellipsoid to clavate, some subglobose, 47–60 × 22–31 μm	Fusoid, 3-septate, constricted, 18–25 × 6–8 μm	Yang et al. (2021)
*T. ornatum*	125–250 μm in diam.	Straight or curved, septate, 58–120 × 5–9 μm	–	Cylindric-fusoid, 3-septate, not constricted, 22–27 × 6–10 μm	Chomnunti et al. (2012a)
*T. pelliculosum*	80–145 μm in diam.	Straight or curved, 54–95 × 5–10 μm	–	Fusoid, 1–3 septate, hyaline, not constricted, 15–22 × 4–7 μm	Chomnunti et al. (2012a)
*T. siamensis*	112–130 μm in diam.	Curved, 38–56 × 4–5 μm	Cylindrical or oblong, bitunicate, short pedicel, 48–64 × 14–21 μm	Oblong to ellipsoid, 3-septate, not constricted, 20–23 × 6–7 μm	Liu et al. (2015)
*T. xishuangbannaense*	94–117 × 89–112 μm	Straight, abundant, aseptate, 65–121 × 4–7 μm	Obovoid to ellipsoid, bitunicate, 42–58 × 22–29 μm	Fusoid, 2–3-septate, not constricted, with many guttules, 23–29 × 6–9 μm	Yang et al. (2021)
*T. xishuiense*	260.4 μm in diam.	Spindle-shaped, blunt ends, abundant, straight, aseptate, 34–41 × 3.7–5 μm	Cylindrical to clavate, bitunicate, 58.74–68.72 × 7.1–14.6 µm	Fusoid, 1–2-septate, constricted, with guttules, 15.6–26.3 × 4.5–5.5 μm	The study
*T. yunnanense*	–	Abundant, straight, aseptate, 80–118 × 4.3–7.2 μm	Long ellipsoid, bitunicate, 59–69 × 18–23 μm	Long ellipsoid, 2–3 septate, with guttules, 19–24 × 6.5–8.4 μm	Yang et al. (2021)

‘ – ‘ indicates a missing detailed description in the original literature.

#### Trichomerium xishuiense

Taxon classificationFungiChaetothyrialesTrichomeriaceae

X. L. Deng & X. Y. Zeng sp. nov.

6E275633-C280-5CB3-A13E-48FB36FBF30D

Fig. 3

##### Etymology.

In reference to the location where the specimen was collected

**Figure 3.**
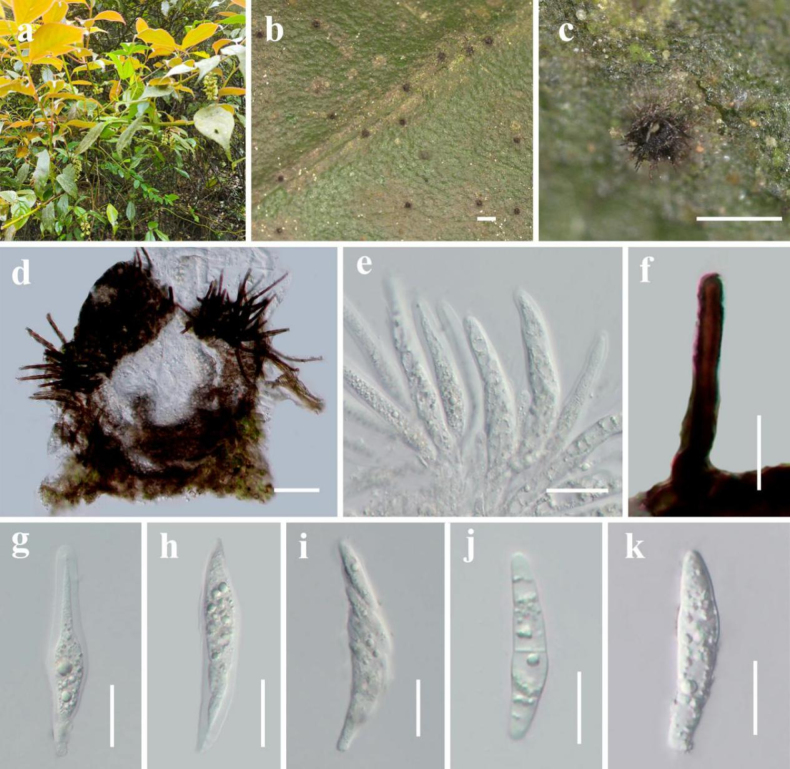
*Trichomerium xishuiense* (GUCC 23-0131). **a, b**. Sooty moulds on leaf surface; **c, d**. Ascomata with setae; **e**. Asci; **f**. Seta; **g–i**. Asci; **j, k**. Ascospores. Scale bars: 500 µm (**b**), 250 µm (**c**), 50 µm (**d**), 20 µm (**e–k**).

##### Description.

Black sheets of superficial mycelia cover the surface of leaves of the host. Hyphae are septate, cylindrical, light brown to brown. **Sexual morph**. Ascomata up to 260.4 µm in diameter, subglobose to globose, sessile, with an ostiole, with scattered setae on the upper surface. Setae 42.74–57.49 µm long (x̄= 50.39 µm, n = 10), dark brown or olive. Asci 58.74–68.72 × 7.1–14.6 µm (x̄= 64.35 × 9.97 µm, n = 10), bitunicate, 8-spored, cylindrical to clavate. Ascospores 15.6–26.3 × 4.5–5.5 µm (x̄= 19.35 × 4.5 µm, n = 10), spindle-shaped, with bluntly rounded ends, slightly, transparent, arranged in overlapping patterns, 1–2-septate, constricted at septa, with guttules, without mucous sheath. **Asexual morph**. Undetermined.

##### Material examined.

China • Guizhou Province, Xishui County, 28°29'50"N, 106°23'24"E, 14 April 2023, Yuwei Liu, XS3J (HGUP 23-0080, holotype), ex-type, GUCC 23-0131.

##### Notes.

*Trichomerium xishuiense* is characterized by a combination of traits common in the genus, such as setose ascomata and bitunicate asci with hyaline ascospores. The phylogenetic tree constructed based on ITS and LSU gene sequences (Fig. 1) indicates that strain GUCC 23-0130 forms an independent branch cluster within the *Trichomerium* clade with high statistical support (ML/BI = 100/1). Phylogenetically, *Trichomerium* is also close to *Formicomyces* and *T. bambusae*, forming sister groups with each other. Compared to its closest sister species, *T. bambusae*, the two share 11% sequence divergence in the ITS region (379/426 bases, including 3 gaps). Despite this phylogenetic affinity to *Formicomyces*, *T. xishuiense* exhibits the characteristic sexual morph with setose ascomata and bitunicate asci, aligning it morphologically with the genus *Trichomerium* rather than the asexual, morphologically distinct genus *Formicomyces*. *Formicomyces* has been found only in its asexual phase to date. Its most distinctive feature is the apical modification of the conidiophore into a funnel-shaped or cup-like collar structure, which produces single-celled, morphologically simple, elliptical to lemon-shaped conidia (Igor et al. 2023). The genus *Trichomerium*, to which *T. xishuiense* belongs, is not only known for its sexual phase but also possesses a distinctive asexual phase. This phase typically produces dark-colored, structurally complex, multi-segmented conidia that aggregate into unique multi-armed stellate structures. These clear morphological evidences confirm that *T. xishuiense* should be classified within the genus *Trichomerium* rather than *Formicomyces*. Moreover, *T. xishuiense* differs from its sister species *T. bambusae* in its larger ascomata (up to 260.4 µm in diam. vs. 180 × 105–130 µm), its sessile and larger asci (58.74–68.72 × 7.1–14.6 µm vs. 53–62 × 19–24 µm), and its 1–2-septate, constricted ascospores (vs. 3-septate and non-constricted). Therefore, based on its unique phylogenetic position, significant molecular differences, and clear morphological and ecological distinctions from closely related species, *T. xishuiense* is a new species.

## Discussion

In this study, we describe *T. guiyangense* and *T. xishuiense* as novel species from Guizhou, China. These discoveries expand the known diversity of *Trichomerium* and provide valuable molecular data for future phylogenetic studies within the family.

The genus *Trichomerium* exhibits both teleomorphic and anamorphic stages. The two species identified in this study represent their teleomorphs. Several species within this genus were originally described without molecular sequence data. In the present study, the new species *T. xishuiense* was found to occupy a phylogenetic position outside the core *Trichomerium* clade, adjacent to *Formicomyces*, suggesting the possibility that it may not belong to *Trichomerium*. However, based on morphological characteristics, *T. xishuiense* exhibits typical features of *Trichomerium*, leading to its placement within this genus.

In addition, diagnostic features for distinguishing the teleomorph of *Trichomerium* include the size of developing ascomata, the dimensions and abundance of setae, the presence or absence of septa, and the size and shape of asci, as well as the morphology, size, number of septa and guttules, and presence of a gelatinous sheath of the ascospores (Table 3). Although both *T. gloeosporum* and *T. multisetosum* possess numerous straight setae, the former has larger ascomata (116–140 × 113–150 μm) and ascospores with a distinct gelatinous sheath, whereas the latter has sparser setae and slightly smaller ascomata (116–130 × 91–118 μm). However, due to the high degree of overlap in morphological characteristics among many species within this genus (e.g., ascomata size, the shape and size of the asci, setae distribution, and number of ascospore septa), such as the high similarity in ascus and ascospore morphology between *T. deniqulatum* and *T. multisetosum*, differentiation based solely on morphological features is challenging and may introduce further uncertainty. Therefore, future studies should focus on supplementing molecular sequences for this genus to enable reliable comparative analyses and support integrative taxonomic interpretations.

This study contributes to the taxonomic understanding of the genus *Trichomerium* through detailed morphological descriptions and molecular sequences of two new species—*T. guiyangense* and *T. xishuiense*. Nevertheless, significant gaps remain in the classification of this genus and its associated foliar fungal communities. The current molecular sequence databases are notably incomplete, as many previously described species – including the type species of the genus – lack reference sequences for key genetic markers such as ITS and LSU. Furthermore, the integration of morphological characteristics with molecular data has not been systematically pursued. To address these limitations, future research should prioritize expanding molecular marker coverage with an emphasis on multi-locus sequencing, developing a unified taxonomic framework that combines morphological and molecular evidence, and establishing a more robust and comprehensive phylogenetic system. Advancements in these areas will greatly enhance our understanding of the biodiversity patterns and evolutionary history of *Trichomerium* and its related families.

## Supplementary Material

XML Treatment for Trichomerium guiyangense

XML Treatment for Trichomerium xishuiense

## Additional information

### Use of AI

No use of AI was reported.

### Author ORCIDs

Xiaolu Deng https://orcid.org/0009-0003-3475-0096

Yuwei Liu https://orcid.org/0000-0002-0346-149X

Sinang Hongsanan https://orcid.org/0000-0003-0550-3152

Yuzhe Feng https://orcid.org/0009-0000-8832-7355

Jipeng Sun https://orcid.org/0009-0004-0065-6920

Xiang-Yu Zeng https://orcid.org/0000-0003-1341-1004

### Data availability

All of the data that support the findings of this study are available in the main text.
